# Inhalation exposure to nanosized and fine TiO_2 _particles inhibits features of allergic asthma in a murine model

**DOI:** 10.1186/1743-8977-7-35

**Published:** 2010-11-25

**Authors:** Elina M Rossi, Lea Pylkkänen, Antti J Koivisto, Heli Nykäsenoja, Henrik Wolff, Kai Savolainen, Harri Alenius

**Affiliations:** 1 Unit of Excellence for Immunotoxicology, Finnish Institute of Occupational Health, Helsinki, Finland; 2 New Technologies and Risks, Finnish Institute of Occupational Health, Helsinki, Finland; 3 Biological Mechanisms and Prevention of Work-Related Diseases/Immunopathology, Finnish Institute of Occupational Health, Helsinki, Finland

## Abstract

**Background:**

Nanotechnology and engineered nanomaterials (ENM) are here to stay. Recent evidence suggests that exposure to environmental particulate matter exacerbates symptoms of asthma. In the present study we investigated the modulatory effects of titanium dioxide particle exposure in an experimental allergic asthma.

**Methods:**

Nonallergic (healthy) and ovalbumin-sensitized (asthmatic) mice were exposed via inhalation to two different sizes of titanium dioxide particles, nanosized (nTiO_2_) and fine (fTiO_2_), for 2 hours a day, three days a week, for four weeks at a concentration of 10 mg/m^3^. Different endpoints were analysed to evaluate the immunological status of the mice.

**Results:**

Healthy mice elicited pulmonary neutrophilia accompanied by significantly increased chemokine CXCL5 expression when exposed to nTiO_2_. Surprisingly, allergic pulmonary inflammation was dramatically suppressed in asthmatic mice which were exposed to nTiO_2 _or fTiO_2 _particles - i.e. the levels of leucocytes, cytokines, chemokines and antibodies characteristic to allergic asthma were substantially decreased.

**Conclusions:**

Our results suggest that repeated airway exposure to TiO_2 _particles modulates the airway inflammation depending on the immunological status of the exposed mice.

## Background

The exploding market of nanobased products and nanotechnology as a whole have put the health professionals and regulatory authorities at an alert. There is already growing evidence on the potential adverse health effects on healthy individuals, but only part of the world's population can be categorized into this group. A large part of the population has impaired health conditions that will make them more susceptible to develop health problems from particulate exposure.

In industrialized countries asthma and allergies are increasingly prevalent. According to the European Academy of Allergy and Clinical Immunology (EAACI) one in three children today is allergic and 30-50% of them will develop asthma. It is estimated that by year 2015 half of all Europeans may be suffering from allergy [1]. Asthma is a product of both genetic predisposition and environmental conditions. Children in wealthy countries are more likely to develop allergy-related asthma than children in poorer nations [2]. Hygiene hypothesis suggests that lack of intense infections due to improved hygiene, vaccination and antibiotics has altered the immune system to improperly respond to neutral substances [3]. Approximately 80% of asthma cases today are caused by allergies. Evidence already exists that environmental particulate matter, such as air pollutants and diesel exhaust particles, enhances airway hyperresponsiveness and exacerbation of asthma as well as increases respiratory and cardiovascular mortality and morbidity [4-6]. The most susceptible population groups for these adverse health effects include elderly subjects with chronic cardiorespiratory disease, as well as children and asthmatic subjects of all ages.

Nanosized and larger particles of titanium dioxide (TiO_2_) are widely used in many fields of science and technology. According to the IARC [7] titanium dioxide accounts for 70% of the total production volume of pigments worldwide and is classified as possibly carcinogenic to human beings (ie, group 2B). TiO_2 _is used in various applications such as paints, coatings, UV protection, photocatalysis, sensing and electrochromics, photochromics as well as food colouring [8]. Brightness and high refractive index are properties that have made TiO_2 _the most widely used white pigment. Other properties of TiO_2 _include chemical stability, low toxicity and cheap price. Plain TiO_2 _nanoparticles are often altered to better and more specifically suit their uses. Alterations can be made by doping TiO_2 _with other elements or by modifying the surface with other semiconductor materials. TiO_2 _mostly occurs as rutile, anatase or brookite chrystalline polymorphs.

In the present study we explored the effects of repeated inhalation exposure in asthmatic and healthy mice to two different sizes of TiO_2_. We demonstrate that contrary to expectations exposure to fine or nanosized particles inhibits most soluble and cellular mediators of allergic asthma. The present study emphasizes that it is crucial to take into account the heterogeneity of the state of health of individuals in assessing health implications of nanoparticle exposure in humans.

## Materials and methods

### Test materials

**Figure 1:**
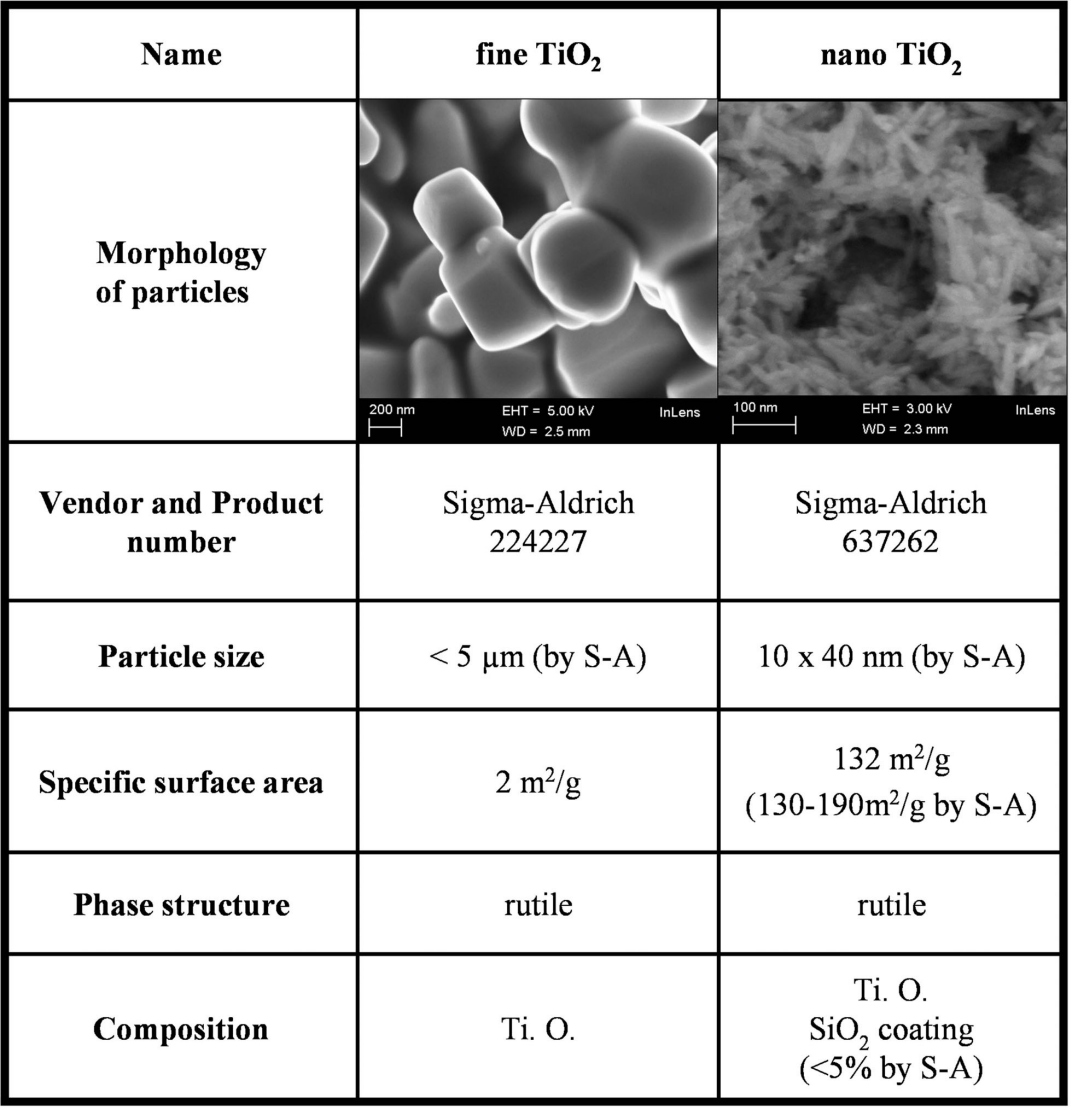
**Particle characteristics**. Particles used in this study are listed in this table. All characteristics are measured by us as described in the materials and methods section unless stated otherwise in the table. Pictures of nanoparticles are taken using scanning electron microscopy[9].

Two different types of titanium dioxide (TiO_2_) particles were used in our experiments. The other TiO_2 _was nanosized and the other coarser fine-sized. The fine rutile particle (fTiO_2; _product number 224227, Sigma-Aldrich, Steinheim, Germany) had an initial particle size of under 5 μm and nanosized rutile (nTiO_2_; product number 637262, Sigma-Aldrich) was silica coated, needle-like and ca. 10 × 40 nm in size. Both materials were thoroughly characterized before and during exposures (Figure 1, [9]). The size and morphology of the nanopowders were characterized by electron microscopy (Zeiss ULTRAplus FEG-SEM, Carl Zeiss NTS GmbH, Oberkochen, Germany and JEM 2010 TEM, Jeol Ltd., Tokyo, Japan) and their composition by energy dispersive spectroscopy (EDS; ThermoNoran Vantage, Thermo Scientific, Breda, The Netherlands). The crystallinity and phase composition of the nanopowders were characterized by powder x-ray diffraction analysis (Siemens D-500, Siemens AG, Kahrlruhe, Germany) and specific surface area of nanopowders was measured by nitrogen adsorption, using the Brunauer-Emmett-Teller method (Coulter Omnisorp 100CX, Florida, USA).

### Exposure system for aerosolized particles

The animals were exposed using a solid particle dispenser (Rotating Brush Generator, RBG 1000, Palas, Karlsruhe, Germany) as described earlier by Rossi *et al*. (2010).

### Aerosol and particle characterization

**Figure 2:**
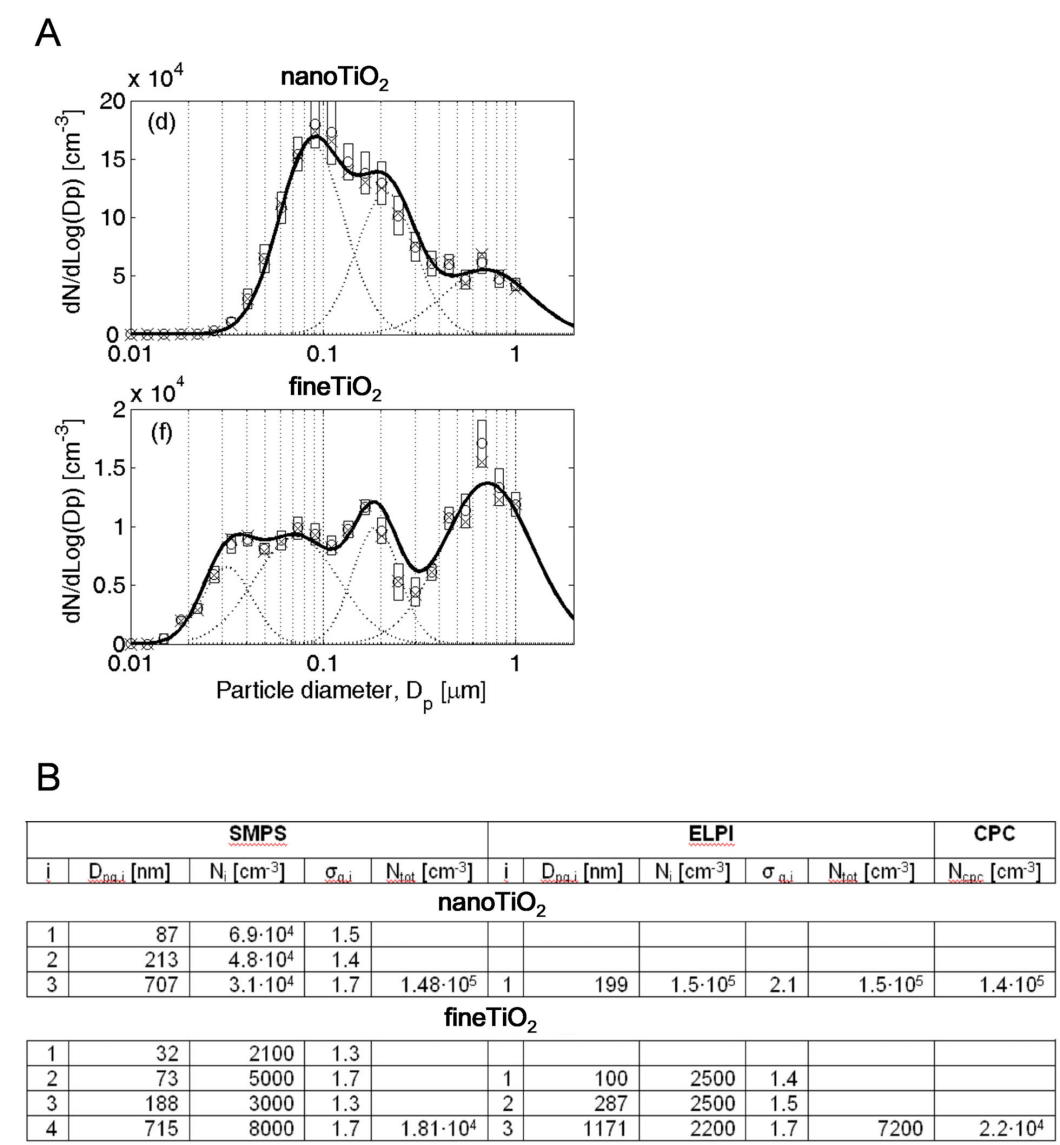
**Particle and aerosol characteristics**. **A) **Particle mobility and aerodynamic number size distributions measured with SMPS. Size bin concentrations are represented as mean (o), median (x) and standard deviation (box). Solid line represents mean of multi log-normal function and dotted lines are means of single modes. Measurements and fitting were done during steady-state condition. **B) **Multi log-normal fit parameters for Fig 2A. particle size distributions and mean particle concentration measured by CPC [32]. D_pg, i_, N_i _and σ_g, i _are geometric mean diameter, particle concentration and geometric standard deviation of mode i, respectively, and N_tot _is total particle concentration of size distribution.

The aerosol size distributions were measured from 10 nm to 1000 nm with a scanning mobility particle sizer (SMPS) consisting of a ^63^Ni bipolar aerosol neutralizer, a Vienna type differential mobility analyzer (DMA) with length of 28 cm and TSI model 3010 condensation particle counter (CPC) [10]. Aerosol aerodynamic size distribution was measured from 15.9 nm to 10 μm with an electronic low pressure impactor (ELPI, Dekati) (Figure 2) [11]. The aerosol mass concentration was determined gravimetrically after collection on nitrocellulose filters (Millipore). Particle concentration inside the particle dispenser chamber was also monitored using a personal DataRAM (online mass concentration meter, *p*DR1000AN, Waltham, MA, USA).

### Animals - sensitization and exposure protocol

Following a 1-week acclimation period, 7-week-old female BALB/c/Sca mice (Scanbur AB, Sollentuna, Sweden) were randomized into two exposure and control groups (8 mice/group). Mice were sensitized intraperitonally with 20 μg of ovalbumin (OVA) in alum (Sigma-Aldrich, St Louis, MO) in 100 μl of phosphate-buffered saline (PBS) on days 1 and 14 of the experiment. Control group was given alum in 100 μl of PBS. Exposure groups were exposed three times a week for 2 hours for the duration of the four week experiment. The exposure concentration was 10 ± 2 mg/m^3 ^in all tests. This concentration was chosen to mimic occupational conditions where workers are exposed to concentrations of around 5 mg/m^3 ^[12]. On days 25-27 all mice were challenged with 1% OVA solution via the airways for 20 min administered using the ultrasonic nebulizer (DeVilbiss, Glendale Heights, IL).

### Measurement of airway responsiveness

Airway responsiveness was measured on day 28 using a single chamber, whole-body plethysmograph system (Buxco, Troy, NY) as described earlier [13]. Briefly, mice were exposed to increasing concentrations (1, 3, 10, 30 and 100 mg/ml) of metacholine (MCh; Sigma Aldrich) in PBS delivered via an AeroSonic 5000 D ultrasonic nebulizer (DeVilbiss, ITW, Glendale Heights, IL). Before MCh exposure the baseline is measured for three minutes. After baseline measurements the MCh is nebulized for 1.5 minutes and airway reactivity is measured for 5 minutes per concentration. Lung reactivity parameters were expressed as Penh (enhanced pause) values. After measurement of lung responsiveness, the mice were sacrificed using an overdose of isoflurane and samples were collected for analysis.

### Collection of biological samples and preparation

Sample collection and preparation were done as described previously by Rossi *et al*. (2010). The following samples were collected: blood serum for antibody analysis, bronchoalveolar lavage (BAL) cells and supernatant for May Grünwald-Giemsa (MGG) staining and protein analysis and lung samples for RNA isolation and hematoxylin and eosin (H&E) and Periodic Acid-Schiff (PAS) staining. The spleens were also dissected from the mice onto 6-well plates with PBS for spleen cell stimulations.

### Spleen cell stimulations

Spleens were mechanically broken down into paste and filtered to remove larger pieces. Cells were resuspended in RPMI media, counted under light microscopy and plated at a concentration of one million cells/ml. Cells to be stimulated were plated in RPMI containing 100 μg/ml ovalbumin. All cells were incubated for 48 hours after which the supernatant was collected and stored at -70°C prior to analysis.

### mRNA and protein analyses

#### RNA isolation from the lung tissues

The lung samples were homogenized in a FastPrep FP120 (BIO 101, Thermo Savant, Waltham, Mass. USA) -machine and RNA was extracted using the FastRNA Pro Green Kit (Qbiogene/MP Biomedicals, Illkirch, France) and its instructions.

#### cDNA synthesis

cDNA was synthesized from 1 μg of total RNA in a 25 ul reaction using MultiScribe Reverse Transcriptase and random primers (The High-Capacity cDNA Archive Kit, Applied Biosystems, Foster City, CA) using the manufacturer's protocol. The synthesis was performed in a 2720 Thermal Cycler (Applied Biosystems, Carlsbad, California, USA) starting with 25°C for 10 minutes and continuing with 37°C for 120 minutes.

#### Polymerase chain reaction (PCR) amplification

PCR primers and probes were ordered as pre-developed assay reagents (18 S rRNA, TNFα, IL-1β, IL-4, IL-13, IL-10, foxp3, CCL3, CXCL5 and CXCL2) from Applied Biosystems (Carlsbad, California). The real-time quantitative PCR was performed as described previously by Rossi *et al*. (2010).

#### Enzyme-linked immunosorbent assay (ELISA)

TNF-α and IL-13 protein levels from spleen cell supernatants were determined using ELISA kits (eBioscience, San Diego, CA, US) used according to manufacturer's instructions. An ELISA plate microtiter reader (Multiskan MS, Labsystems, Helsinki, Finland) was used to read the results.

#### Luminex

For analysis of TNF-α and IL-13 proteins in BAL fluid supernatants we used a Milliplex Mouse Cytokine/Chemokine Immunoassay (Millipore Corporation, Billerica, MA) according to the manufacturers' protocol. 3% bovine serum albumin (BSA; Sigma-Aldrich, St Louis, MO) in PBS was added at a concentration of 0.5% to samples, controls and standards to ensure sufficient protein amounts for the assay. Assay was performed using Luminex xMAP Technology (Bio-Plex 200 System, BioRad, Hercules, CA).

### Measurement of serum antibodies

For the analysis of OVA-specific IgE, ELISA plates were coated with Purified Rat Anti-mouse IgE antibody (BD Biosciences, San Jose, CA) and incubated overnight. On the second day the plates were blocked with 3% BSA in PBS, serum samples (1:10 and 1:20 dilution) were added to the plate and incubated overnight. Biotinylated OVA was added and streptavidin horseradish peroxidase (Pharmingen 13047E, BD Biosciences) followed by ABTS solution (ABTS Microwell Peroxidase Substrate System, KPL, Gaithersburg, MD). Absorption was read at 405 nm with ELISA plate absorbance reader (Multiskan MS, Labsystems, Helsinki, Finland).

For OVA-specific IgG2a analysis, ELISA plates were coated with ovalbumin (BD Biosciences) and incubated overnight. On the second day the plates were blocked with 3% BSA in PBS, serum samples (1:60, 1:180, 1:540 and 1:1620 dilution) were added to the plate and incubated overnight. Biotinylated anti-mouse IgG2a (Pharmingen 02012 D, BD Biosciences) was added and streptavidin horseradish peroxidase (BD Biosciences). ABTS solution and peroxidase were mixed 50:50 and added before reading absorption at 405 nm with ELISA plate absorbance reader.

### Analysis of mucus producing cells

The lung tissue was analysed from formalin-fixed, paraffin-embedded sections. These sections were stained for mucus secreting goblet cells using Periodic acid-Schiff (PAS) -stain. The data was analyzed with Leica Image Manager IM50 version 4.0 (Leica Microsystems Imaging Solutions Ltd., Cambridge, UK). PAS+ cells were counted as an average of PAS+ cells found in 100 μm of bronchus counted from three bronchioles of similar size per mouse (n = 8 mice per group).

### Hydroxyl radical (^•^OH) formation capacity

The ^•^OH formation capacity was determined using the benzoic acid probe as described earlier by Rossi *et al*. (2010).

### Statistical analysis

The toxicological data was analyzed with the GraphPadPrism software (GraphPadPrism Software, Inc., San Diego, CA). For all statistical analysis we first performed analysis of variance using one-way ANOVA (nonparametric Kruskal-Walles test) and when the ANOVA was positive we performed post-testing. The different mice groups were compared using nonparametric Mann-Whitney U - test. P-values less than 0.05 were considered statistically significant.

## Results

### Inhalation exposure to TiO_2 _particles reduces leukocyte numbers and mucus secretion in the airways of asthmatic mice

**Figure 3:**
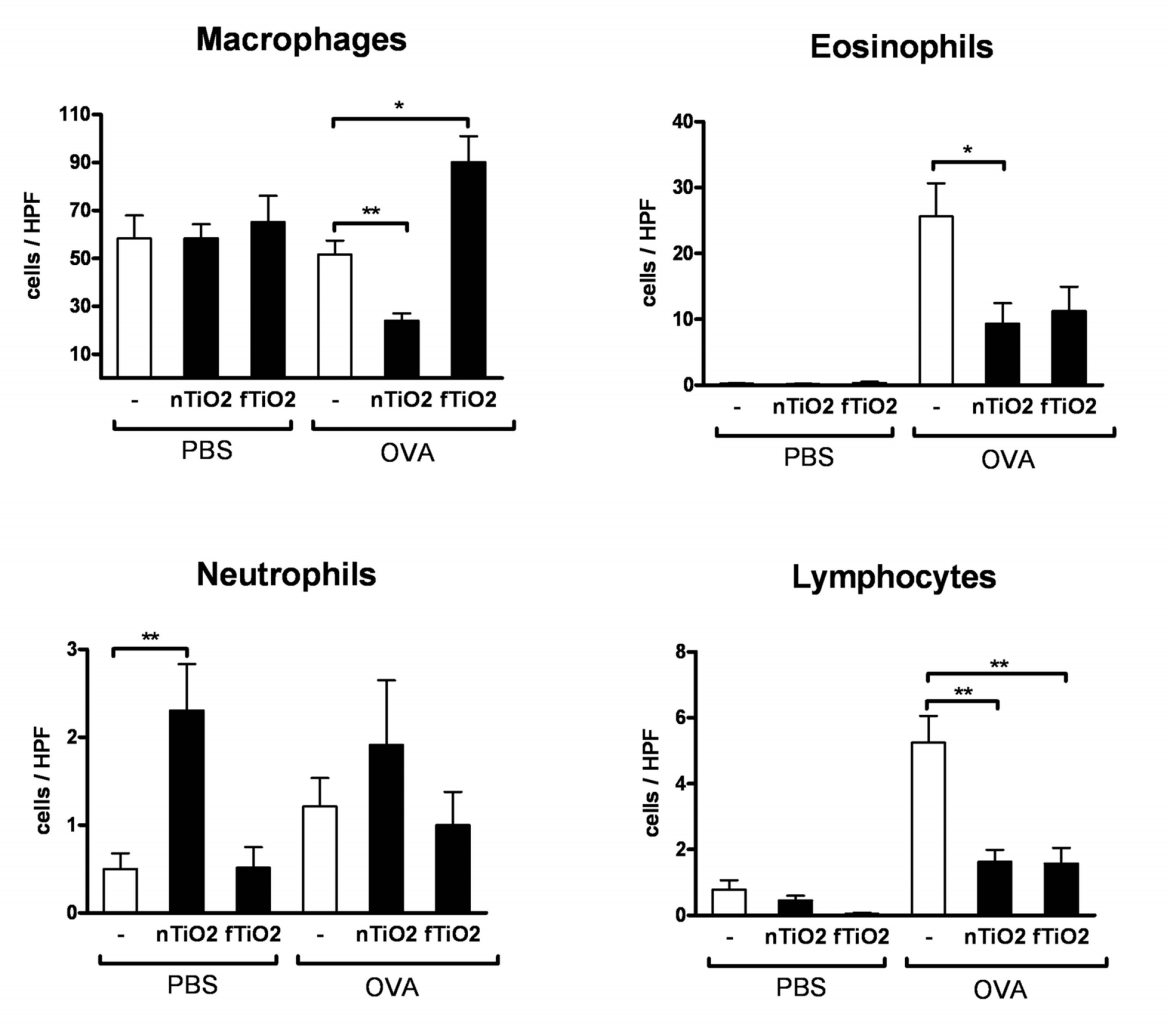
**Cell count in bronchoalveolar lavage fluid**. The effect of titanium dioxide (TiO_2_) inhalation exposure performed for 2 hours a day, three days a week, for four weeks at a concentration of 10 mg/m^3 ^on macrophage, eosinophil, neutrophil and lymphocyte infiltration to bronchoalveolar lavage fluid (BALF) calculated from May-Grünwald-Giemsa (MGG)-stained cytospin slides with light microscopy (x80). Results are shown as cells per high power field (HPF) for healthy (PBS) and allergic (OVA) mice exposed to nanosized (nTiO_2_) or fine (fTiO_2_) TiO_2 _or left unexposed (-). From each slide the cells were counted from three fields from which an average was counted. N = 8 mice per group. The bars represent mean ± SEM; * P < 0.05 and ** P < 0.01 significantly different from unexposed control; Mann-Whitney U test.

Asthmatic mice demonstrate increased numbers of eosinophils and lymphocytes in the airways compared to healthy controls. In healthy mice (PBS) there was a significant 4.6-fold increase in the influx of neutrophils following exposure to nTiO_2 _(Figure 3) whereas the numbers of macrophages, eosinophils and lymphocytes remained unaffected. Exposure to fTiO_2 _had no effects on any cells in the BAL of healthy mice. Surprisingly, the numbers of eosinophils and lymphocytes, characteristic features of allergic inflammation, were dramatically reduced in asthmatic mice (OVA) after exposure to both nTiO_2 _and fTiO_2_. Macrophages were reduced in half following nTiO_2 _and increased 1.7-fold following fTiO_2 _exposure in asthmatic mice. Changes in numbers of pulmonary neutrophils remained nonsignificant.

**Figure 4:**
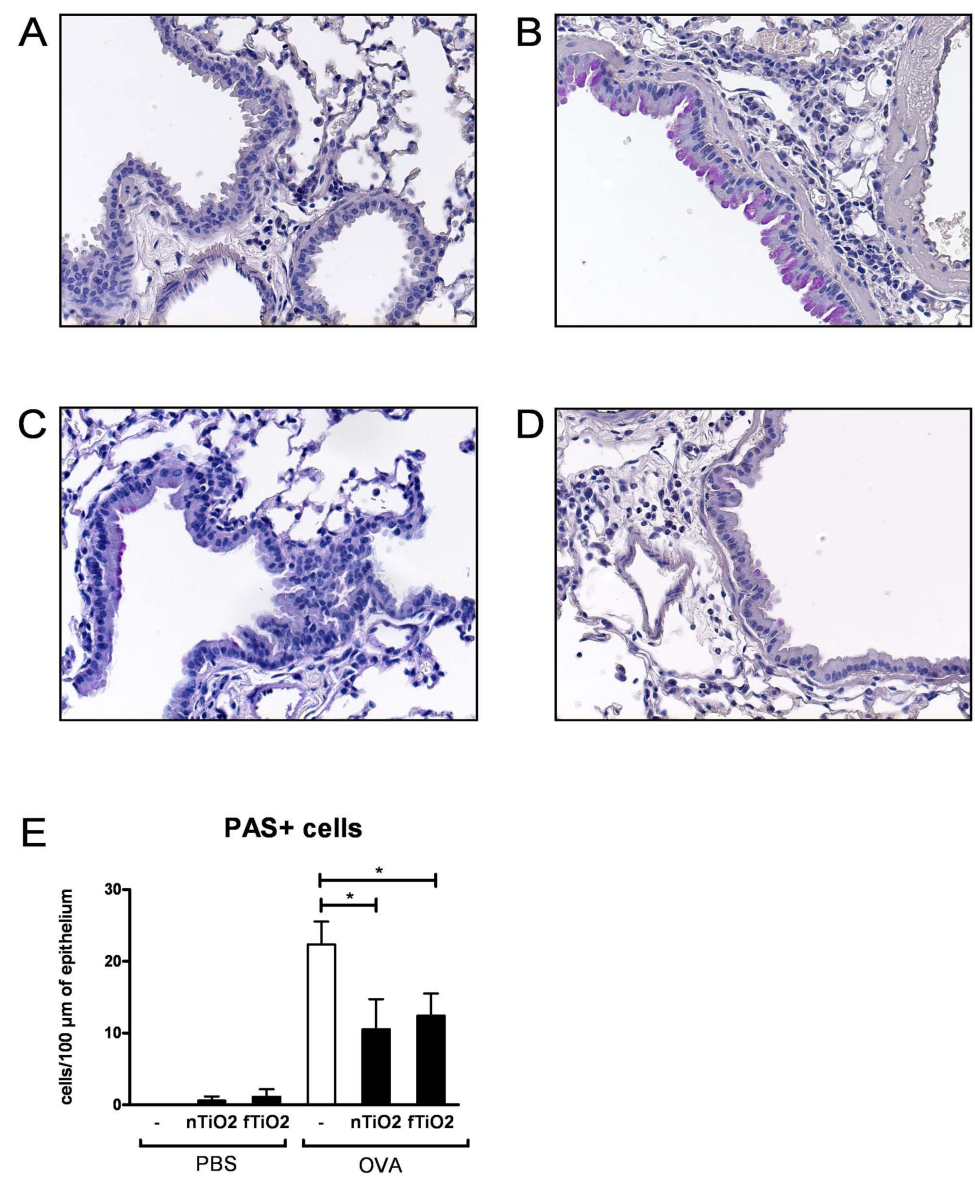
**Occurence of mucus producing cells in bronchioles**. Periodic acid-Schiff (PAS) -stained mouse lung tissue, where mucus producing goblet cells can be seen in colour red around the bronchioles. Tissue samples from unexposed healthy (A) and asthmatic (B) mice and asthmatic mice exposed to nanosized (C; nTiO_2_) or fine (D; fTiO_2_) TiO_2_. (E) Effects of titanium dioxide exposure on the amount of mucus-producing cells around the bronchioles. Results are indicated as the average of PAS+ cells/100 μm of bronchus counted from three bronchioles per mouse (n = 8 mice per group) for healthy (PBS) and allergic (OVA) mice exposed to nanosized (nTiO_2_) or fine (fTiO_2_) TiO_2 _or left unexposed (-). N = 8 mice per group. The bars represent mean ± SEM; * P < 0.05 significantly different from unexposed control; Mann-Whitney U test.

Goblet cell hyperplasia in the lungs is a common feature of allergic asthma and mucus secreting PAS+ goblet cells are usually not present in bronchioles and smaller conducting airways of mice [14]. Healthy mice showed clear lungs with no mucus secreting cells (Figure 4A) whereas PAS+ cells were abundant in the bronchial epithelium of allergic mice (Figure 4B). Both TiO_2 _particles caused a drastic reduction in the numbers of PAS+ cells in the allergic mice (Figure 4C nTiO_2 _and D fTiO_2_). Both exposed groups showed a statistically significant decrease in goblet cell numbers in the epithelium (Figure 4E) after the particle exposure.

### Airway reactivity to inhaled metacholine is significantly affected by particle exposure

**Figure 5:**
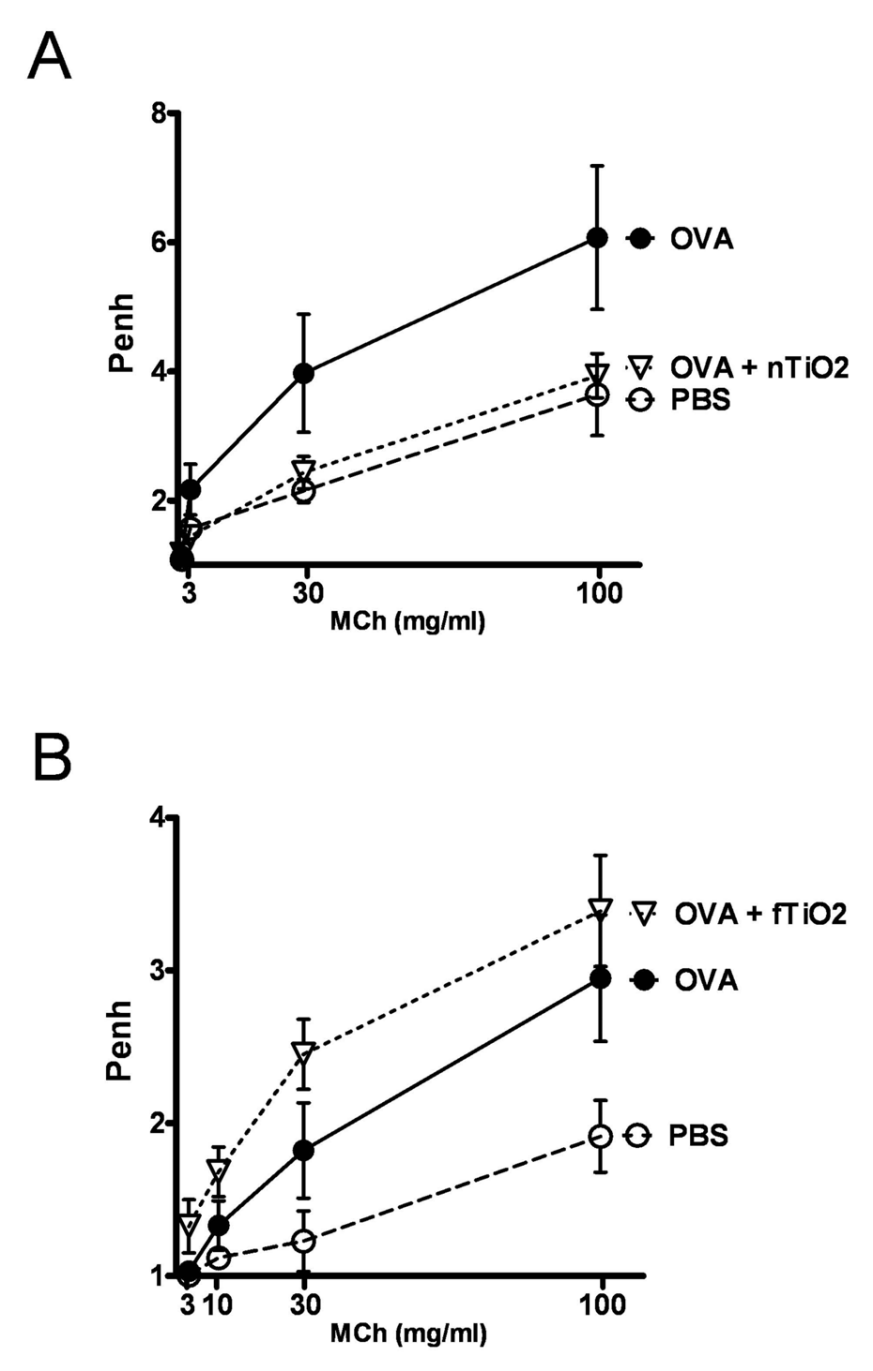
**Airway responsiveness to metacholine**. Effect of airway responsiveness to metacholine (MCh) in allergic mice after exposure to nanosized (A; nTiO_2_) or fine (B; fTiO_2_) TiO_2 _for 2 hours a day, three days a week, for four weeks at a concentration of 10 mg/m^3 ^(n = 8 mice per group). Results are shown as enhanced pause (Penh) values, dimensionless values that we used to empirically monitor airway function, in relation to increasing doses (1-100 mg/ml) of aerosolized MCh for healthy (PBS) and allergic (OVA) mice.

Airway hyperresponsiveness, a hallmark of asthma, was measured by airway reactivity to increased doses of inhaled metacholine (Mch). Exposure of OVA sensitized and challenged mice to nTiO_2 _(Figure 5A) reduced airway hyperreactivity (AHR) to the level of healthy mice. On the contrary, exposure to fTiO_2 _(Figure 5B) slightly increased the reactivity of the lungs, showing modest exacerbation of asthmatic symptoms. We also exposed PBS groups to TiO_2 _particles (data not shown) but no difference on airway reactivity to inhaled metacholine was found between them and unexposed PBS groups.

### Particle exposure results in a dramatic inhibition of proinflammatory, regulatory and Th2 cytokines in the lungs of asthmatic mice

**Figure 6:**
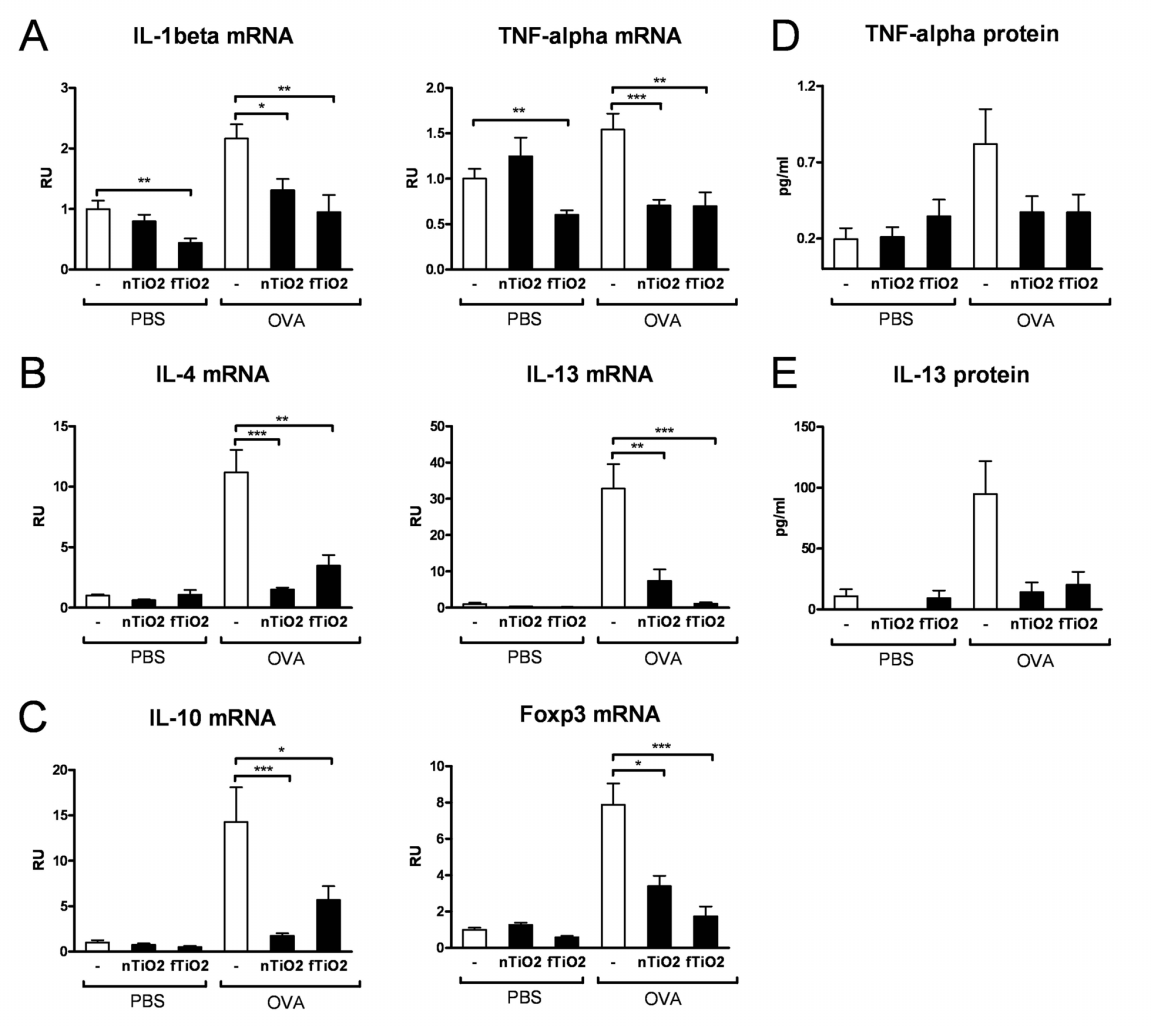
**mRNA and protein expression of cytokines**. Relative mRNA-expression in the lung tissue of (A) proinflammatory cytokines, IL-1β and TNF-α, (B) Th2 type cytokines IL-4 and IL-13, (C) regulatory cytokine IL-10 and marker of regulatory T-cells Foxp3. Protein levels (pg/ml) of proinflammatory cytokine TNF-α (D) and Th2-type cytokine IL-13 (E) in BAL supernatant of mice. Results are shown in relative units (RU) or (pg/ml) for healthy (PBS) and allergic (OVA) mice exposed for 2 hours a day, three days a week, for four weeks at a concentration of 10 mg/m^3 ^to nanosized (nTiO_2_) or fine (fTiO_2_) TiO_2 _or left unexposed (-). N = 8 mice per group. The bars represent mean ± SEM; * P < 0.05, ** P < 0.01, and *** P < 0.001 significantly different from unexposed control; Mann-Whitney U test.

The mRNA expression of proinflammatory cytokines, IL-1β and TNF-α (Figure 6A), was downregulated after particle exposure to about half and third respectively in asthmatic mice when compared to the nonexposed control. IL-1β and TNF-α levels were also reduced in half in healthy (PBS) mice following exposure to fTiO_2_. Similarly, Th2 type cytokines IL-4 and IL-13 (Figure 6B), which are present in asthmatic but not in healthy mice, were significantly diminished after exposure to both nTiO_2 _and fTiO_2_. To examine effects of particle exposure on the regulatory machinery we measured mRNA expression of major suppressive cytokine IL-10 and Foxp3, a marker of regulatory T-cells. fTiO_2 _exposure reduced IL-10 levels by 2.5-fold and Foxp3 levels by 4.5-fold. nTiO_2 _exposure again reduced IL-10 levels by over 8-fold and Foxp3 levels by 2.3-fold (Figure 6C). We also looked at if suppression in cytokine expression could be seen at the protein level. Reduction in the protein levels of both TNF-α (Figure 6D) and IL-13 (Figure 6D) in the BAL from asthmatic mice exposed to TiO_2 _particles was observed with statistical significance (one-way ANOVA) in IL-13 levels.

**Figure 7:**
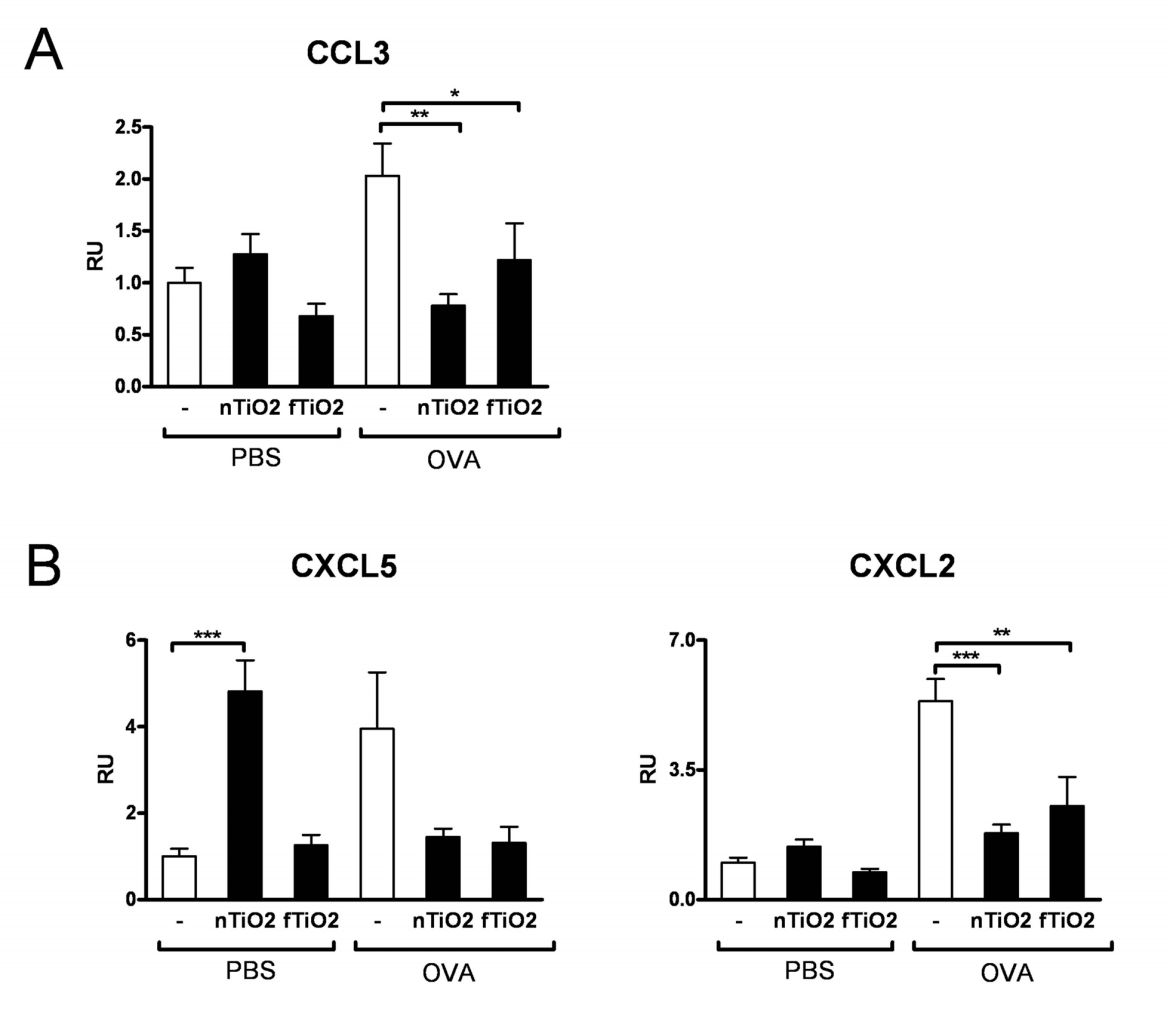
**mRNA expression of chemokines**. mRNA expression of proinflammatory chemokine CCL3 (A) and neutrophil attracting chemokines CXCL5 and CXCL2 (B) in the lung tissue of mice. Results are shown in relative units (RU) for healthy (PBS) and allergic (OVA) mice exposed for 2 hours a day, three days a week, for four weeks at a concentration of 10 mg/m^3 ^to nanosized (nTiO_2_) or fine (fTiO_2_) TiO_2 _or left unexposed (-). N = 8 mice per group. The bars represent mean ± SEM; * P < 0.05, ** P < 0.01, and *** P < 0.001 significantly different from unexposed control; Mann-Whitney U test.

The same suppression phenomenon that was seen in cytokines could be seen with all chemokines examined. mRNA expression of proinflammatory CCL3 (Figure 7A) and neutrophil attracting CXCL5 and CXCL2 (Figure 7B) was decreased in asthmatic mice after exposure to both used particles. In healthy mice exposure to nTiO_2 _caused an almost 5-fold elevation of CXCL5 levels, corresponding to high levels of neutrophils seen in the BALF of the same mice.

### Inhibition of cytokine production in OVA-stimulated spleen cells and reduction in OVA specific IgE levels suggests systemic immune suppression following particle exposure in asthmatic mice

**Figure 8:**
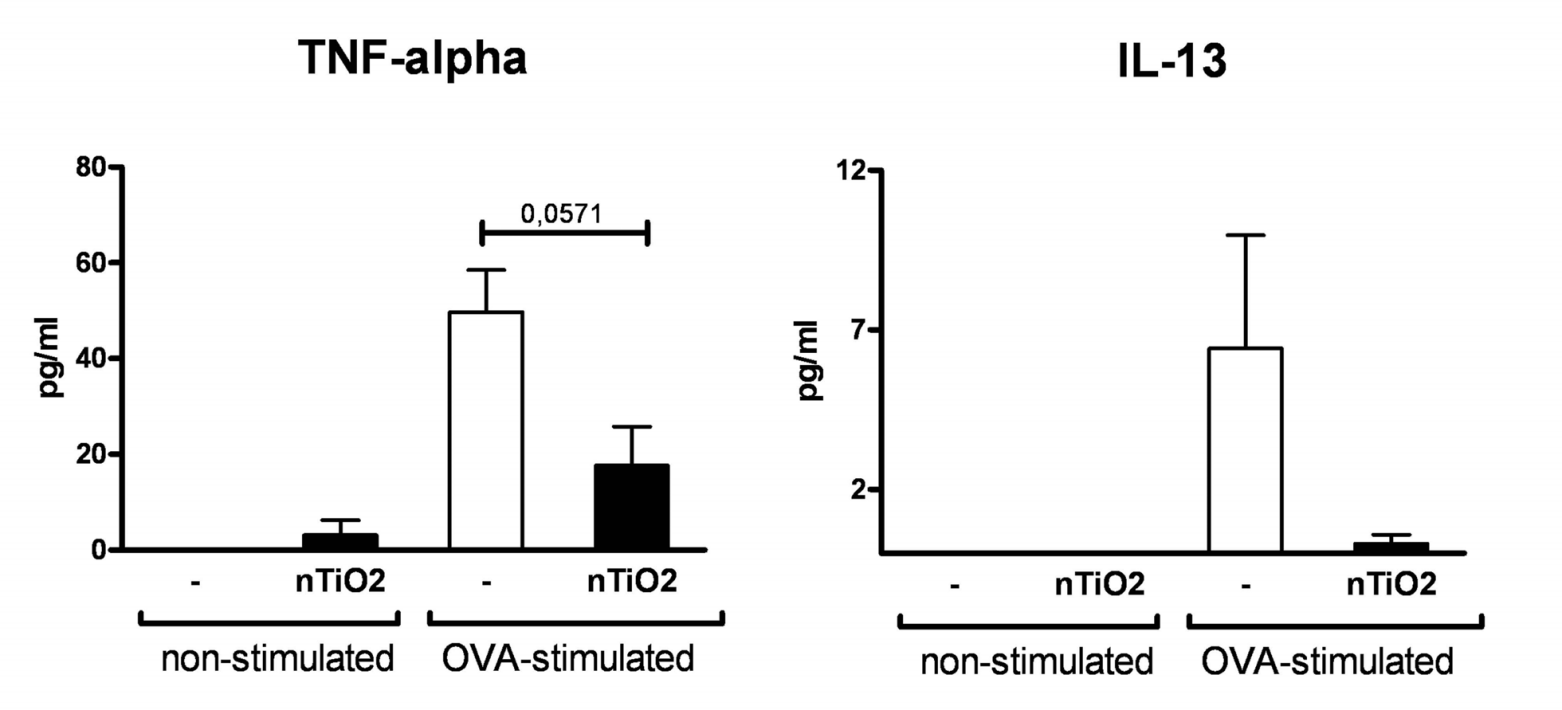
**Protein levels of plated spleen cells**. Protein levels (pg/ml) of TNF-α and IL-13 in the supernatant of plated spleen cells extracted from allergic mice exposed to nTiO_2 _for 2 hours a day, three days a week, for four weeks at a concentration of 10 mg/m^3 ^or left unexposed (-). Cells were either stimulated with OVA or left untreated. N = 8 mice per group. The bars represent mean ± SEM; * P < 0.05 significantly different from unexposed control; Mann-Whitney U test.

To evaluate possible systemic effects of particle exposure we also examined spleen cells from asthmatic nTiO_2 _exposed mice. Spleen cells were either stimulated with OVA or left untreated. OVA stimulated cells express TNF-α and IL-13 as expected, but when the mice had been exposed to nTiO_2 _the protein levels were reduced amply (Figure 8). These results suggest that there is indeed a systemic response that can be shown from the spleen cells.

**Figure 9:**
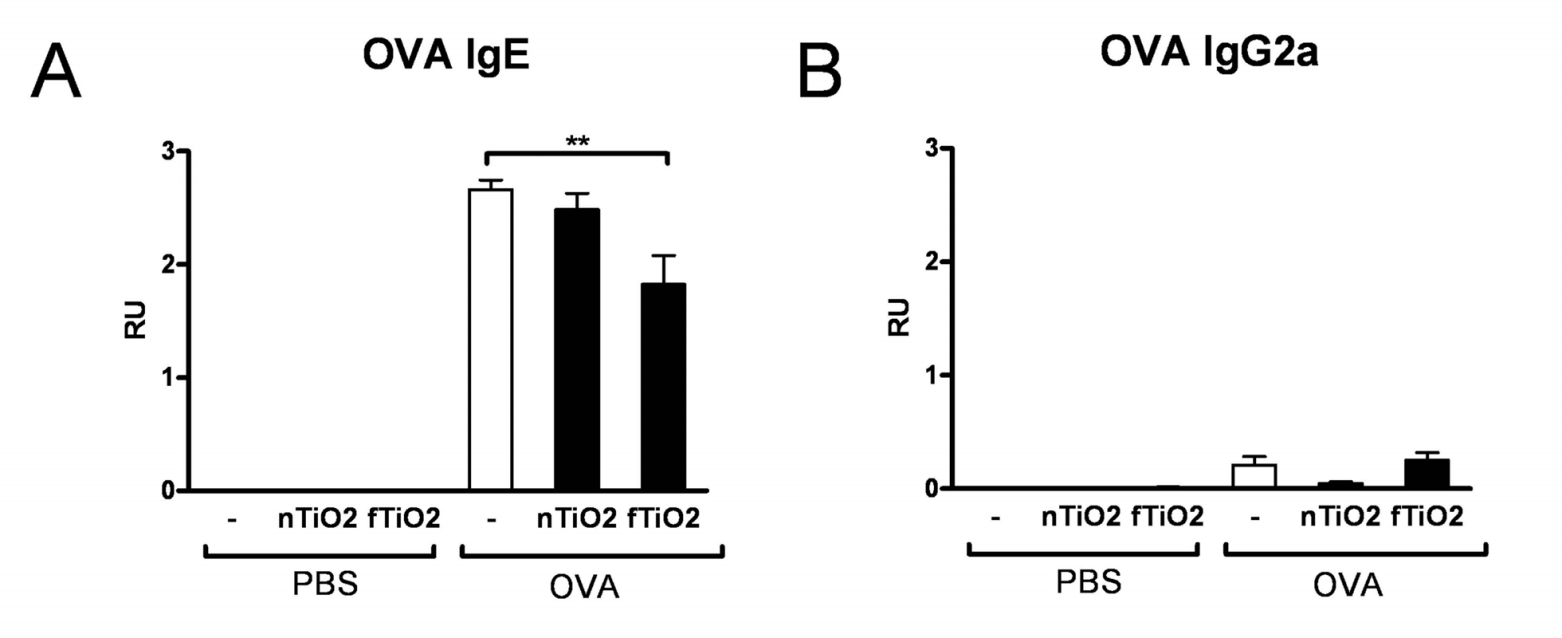
**Expression of antibodies**. Levels of ovalbumin (OVA) specific antibodies IgE (A) and IgG2a (B). Results are shown in relative units (RU) for healthy (PBS) and allergic (OVA) mice exposed for 2 hours a day, three days a week, for four weeks at a concentration of 10 mg/m^3 ^to nanosized (nTiO_2_) or fine (fTiO_2_) TiO_2 _or left unexposed (-). N = 8 mice per group. The bars represent mean ± SEM; ** P < 0.01 significantly different from unexposed control; Mann-Whitney U test.

Increased levels of allergen specific IgE antibodies are characteristic feature of allergic asthma. To investigate effects of particle exposure on circulating antibodies we examined the allergen specific IgE and IgG2a levels. OVA sensitized mice show high levels of OVA specific IgE, but exposure to fTiO_2 _reduces the levels of OVA specific IgE significantly (Figure 9A). On the contrary, no significant changes in the levels of OVA specific IgE could be seen after exposure to nTiO_2 _particles (Figure 9A). Levels of OVA specific IgG2a were low and unaffected by particle exposure (Figure 9B).

### Characteristics of nanoparticles

Initial particle sizes of particles used in this study are for fTiO_2 _< 5 μm and for nTiO_2 _10 · 40 nm as given by the vendor. Aerosolized particles in our experiment occured as agglomerates. Specific surface area of nTiO_2 _is 132 m^2^/g while it's only 2 m^2^/g for fTiO_2 _(Figure 1). The aerodynamic number size distributions indicate that nTiO_2 _dispersed in air using the solid particle dispenser occurred mostly as agglomerates of 100 nm at a number concentration of 69 000 cm^-3 ^whereas fTiO_2 _occurred as agglomerates of 1 μm at a number concentration of 8000 cm^-3 ^(Figure 2A and 2B). fTiO_2 _distribution consist mainly of agglomerates of 0.1 - 2 μm as measured using ELPI. It can be concluded that in particle number concentration most fTiO_2 _are below 1 μm in size but particle mass concentration relies on over 1 μm particles. Analysis of the hydroxyl radical formation capacity using the benzoic acid probe showed that neither of the used particles produced •OH-radicals in a systematic dose-response pattern [9].

## Discussion

We reported recently that repeated inhalation exposure to silica coated rutile titanium dioxide nanoparticles (nTiO_2_) induces pulmonary neutrophilia accompanied by expression of relevant cytokines and chemokines in healthy mice [9]. Out of five different TiO_2 _particles this was the only one that was slightly toxic. The purpose of the present study was to examine immunomodulatory effects of inhalation exposure to different sized particles, nTiO_2 _and fine TiO_2 _(fTiO_2_) in OVA-sensitized, allergic mice. Interestingly, inhalation exposure to both nanosized and fine TiO_2 _caused local and systemic inhibition of several features of experimental asthma. Our results indicate that particle exposure can modulate airway inflammation and airway hyperreactivity in quite distinct ways depending on the immune status of the animals.

Present results obtained from healthy mice were in line with results from our previous study. Exposure to nTiO_2 _particles caused an influx of pulmonary neutrophils and an expression of neutrophil attracting chemokine CXCL5. When healthy mice inhaled fTiO_2 _the mRNA expression of two proinflammatory cytokines, TNF-α and IL-1β respectively, was suppressed. There have been a limited number of relevant studies done with inhalation and the results have been contradictory. Some studies have reported pulmonary neutrophilia after exposure to TiO_2 _nanoparticles whereas others have not [15-20]. Comparison of these ENM is difficult due to different or insufficient methods of particle characterisation. There are also few cases of inhalation exposure reported in humans reporting that inhalation of titanium dioxide may cause metal fume fever [21] or respiratory symptoms accompanied by reduction in pulmonary function [22].

Epidemiological studies have shown that underlying respiratory disease may critically affect the severity of the symptoms when exposed to particulate air pollution [4-6]. The most susceptible population groups for these adverse effects include especially asthmatic subjects of all ages. In the present study we examined whether the asthmatic mice were more susceptible to develop airway inflammation in response to exposure to TiO_2 _nanoparticles when compared to healthy mice. To our surprise exposure to fTiO_2 _and nTiO_2 _significantly downregulated Th2 type inflammation in allergic mice by preventing the infiltration of eosinophils and lymphocytes to the lungs, inhibiting the expression of Th2 cytokines in the BAL as well as decreasing the numbers of mucus producing goblet cells in the airway epithelium. To find out whether the observed suppression was also a systemic phenomenon we stimulated spleen cells from the asthmatic mice with OVA. Stimulated cells derived from asthmatic mice readily expressed TNF-α and IL-13 protein, but cells from asthmatic mice that had been exposed to nTiO_2 _lacked expression of these proteins suggesting that suppression was indeed a systemic effect.

Increased airway hyperreactivity (AHR) to inhaled methacholine (MCh) is one of the hallmarks of allergic asthma. It was of interest that different size TiO_2 _particles acted differently on AHR in the present study. Exposure to nTiO_2 _reduced OVA induced AHR to the baseline level of non-sensitized mice whereas exposure to fTiO_2 _did not have any suppressive effect - actually fTiO_2 _exposed asthmatic mice exhibited slightly higher levels of AHR compared to asthmatic mice without fTiO_2 _exposure. Since IL-13 is known to be closely involved in the development of AHR [23] it can be speculated that reduction of AHR seen in nTiO_2 _exposed mice may at least partly be explained by diminished levels of IL-13 in the airways. On the other hand, fTiO_2 _exposed mice also demonstrated reduced levels of IL-13 mRNA and protein in the airways but their AHR was not suppressed. Thus, yet unidentified factors regulating AHR are likely to play an important role in the modulation of AHR responses during TiO_2 _particle exposure. Th2 cytokines IL-13 and IL-4 also control mucus production and their suppression may explain the clear-cut reduction of goblet cells in the airway epithelia. It is worth mentioning that some differences in the absolute values of Penh were observed in PBS and OVA groups between the two experiments (i.e. Figure 5A and 5B). We want to emphasize that although the scale of Penh values differed between the two experiments, the relative difference between PBS and OVA groups was almost identical. We can therefore quite reliably conclude that the particles studied exhibited different effects on airway reactivity in relation to PBS and OVA groups in these study settings.

Suppression of inflammation similar to the one seen in the present study has been previously reported in the context of exposure to soot and iron oxide [24], oak dust [25], cigarette smoke [26] and fullerenes [27]. Exposure of asthmatic mice to oak dust resulted in suppression of airway reactivity, IL-13, IFN-γ, CXCL5, CXCL2/3 and neutrophils [25]. Thacher *et al*. (2008) reported reduction in eosinophil numbers, PAS+ cells, IL-5, IL-4 and total IgE. Fullerene exposure caused inhibition of anaphylaxis [27]. In contrast to present findings Larsen *et al*. [28] reported that TiO_2 _nanoparticles exhibited a strong adjuvant effect on the development of Th2 dominant immune response in the ovalbumin allergic asthma model. The particles were delivered intraperitonally (IP), which is an unnatural exposure route to nanoparticles in real life. In our study we examined the effects of airway exposure to TiO_2 _particles, which better mimics the real-life exposure scenario. It is important to note that unlike the particles that people more often get exposed to, such as air pollutants and mold, the particles we studied were free of bioactive organic matter. Organic matter contains pathogen-associated molecular patterns that activate innate immunity and cause an enhanced response in allergic mice. In line with this we reported previously [29] that mold exposure together with allergen sensitization caused dramatically enhanced and qualitatively different pulmonary inflammation. The present results demonstrate that exposure to microbe free TiO_2 _particles may not increase Th2 associated airway inflammation and allergic airway hyperreactivity. It should be noted, however, that asthmatic symptoms are not always Th2 associated and therefore responses of exposure to TiO_2 _particles in patients with non-allergic asthma may differ from the present study.

It can be hypothesized that anti-inflammatory Th2 response caused by allergen sensitization may be suppressed by the competing proinflammatory response elicited by TiO_2 _exposure. On the other hand, it has been suggested that T-cell dysfunction results in systemic immune suppression in mice exposed to multiwalled carbon nanotubes [30]. Also in the case of cigarette smoke exposure, reduced T helper function was considered as one possible reason for the suppression of allergic symptoms. Furthermore, consensus exists that Foxp3+ regulatory T-cells are able to control the inflammation thus preventing overactive inflammatory process that harms hosts' own tissue. We therefore also investigated whether the exposure to TiO_2 _particles induces elevated levels of Foxp3, a marker of regulatory T-cells, as well as regulatory cytokine IL-10. Expression levels of Foxp3 and IL-10 were, however, significantly inhibited in asthmatic mice by the particle exposure excluding the possibility that suppression was mediated mainly via regulatory T-cells and regulatory cytokines.

## Conclusions

Assessing risks associated with particle exposure is complicated. This study accentuates that attention has to be paid not only to various characteristics of particles but to various health conditions of people being exposed to them. An interesting study emphasizes these results where pregnancy of healthy mice enhanced lung inflammatory responses to otherwise inert TiO_2 _particles and caused increased allergic susceptibility in their offspring [31]. Very little is known about the physicochemical characteristics which are associated with harmful toxicological potential of nanoparticles. In the present study, the inflammatory potential was not associated with particle size. Even though results slightly varied between the two TiO_2 _particles examined the main mechanism induced by particle exposure in the context of allergic asthma was suppression of Th2 type inflammation. Further studies are needed to provide a better foundation for easier and more reliable assessment and management of risks of nanosized and larger particles.

## Abbreviations

**ABTS**: 2,2'-azino-bis(3-ethylbenzthiazoline-6-sulphonic acid); **AHR**: airway hyperreactivity; **BAL**: bronchoalveolar lavage; **BSA**: bovine serum albumin; **cDNA**: complementary DNA; **CCL3**: C-C motif chemokine 3; **CPC**: condensation particle counter; **CXCL2**: C-X-C motif ligand 2; **CXCL5**: C-X-C motif chemokine 5; **DMA**: differential mobility analyzer; **EAACI**: European Academy of Allergy and Clinical Immunology; **EDS**: energy dispersive spectroscopy; **ELPI**: electronic low pressure impactor; **ENM**: engineered nanomaterials; **foxp3**: forkhead box P3; **fTiO_2_**: fine titanium dioxide; **H&E**: hematoxylin and eosin; **IARC**: The International Agency for Research on Cancer; **IgE**: immunoglobulin E; **IgG2a**: immunoglobulin G, subclass 2a; **IL-1β**: interleukin 1 beta; **IL-4**: interleukin 4; **IL-10**: interleukin 10; **IL-13**: interleukin 13; **MCh**: metacholine; **MGG**: May Grünwald-Giemsa; **nm**: nanometer; **nTiO_2_**: nanosized titanium dioxide; **OH**: hydroxyl radical; **OVA**: ovalbumin; **PAS**: Periodic Acid-Schiff; **PBS**: phosphate-buffered saline; **PCR**: Polymerase chain reaction; **Penh**: enhanced pause; **RPMI**: Roswell Park Memorial Institute medium; **rRNA**: ribosomal ribonucleic acid; **SMPS**: scanning mobility particle sizer; **TNF-α**: tumor necrosis factor-alpha.

## Competing interests

The authors declare that they have no competing interests.

## Authors' contributions

EMR was substantially involved in design of the study, acquisition and analysis of data for all in vivo and in vitro work, statistical analyses, interpretation of results, and drafted the manuscript.

LP was substantially involved in design of the study, interpretation of results, assisted in acquisition of data for in vivo work and revised the manuscript critically. AJK was responsible for the exposure setup and monitoring and characterisation of used particles and aerosols as well as drafting of the manuscript regarding exposure data. HN was substantially involved in acquisition of all in vivo related data. HW was an expert interpreting the histopathological samples in the study. KS was involved in the study as an expert providing expertise on the initial planning of the study and commenting of the manuscript. HA was substantially involved in design of the study, interpretation of results, drafting of the manuscript regarding discussion of data and revised the manuscript critically. All authors have read and approved the final manuscript.
